# Antiparasitic Veterinary Drugs—In Silico Studies of Membrane Permeability, Distribution in the Environment, Human Oral Absorption and Transport Across the Blood–Brain Barrier

**DOI:** 10.3390/membranes16010039

**Published:** 2026-01-10

**Authors:** Anna W. Sobańska, Andrzej M. Sobański, Elżbieta Brzezińska

**Affiliations:** 1 Department of Analytical Chemistry, Medical University of Lodz, Muszyńskiego 1, 90-151 Łódź, Poland; elzbieta.brzezinska@umed.lodz.pl; 2 Faculty of Chemistry, University of Lodz, Tamka 12, 91-403 Łódź, Poland; andrzej.sobanski@edu.uni.lodz.pl

**Keywords:** bioconcentration in fish, biomembrane permeability, blood-brain barrier, veterinary antiparasitic drugs, oral absorption, mobility in soil, QSAR

## Abstract

The present study examined the safety of 86 veterinary antiparasitic drugs in mammals based on their mobility in the soil–water compartment, bioconcentration factor in fish, and blood–brain barrier permeability. An in silico analysis was performed based on biomembrane permeability descriptors, using novel multiple linear regression, boosted tree, and artificial neural network models. Additionally, intestinal absorption in humans was predicted quantitatively using pkCSM software and qualitatively using SwissADME. It was established that the majority of studied drugs are at least slightly mobile in soil, are unlikely to bioaccumulate in fish, and may be absorbed from the human gastro-intestinal tract; in addition, some of them have high potential to enter the mammalian brain.

## 1. Introduction

Veterinary drugs enter the environment by several routes [1], including emissions related to drug manufacture, their use in aquaculture [2,3,4], livestock production (especially industrial-scale farms [5]), natural fertilizers [6,7,8], internal or external application to pet animals [9,10], and the disposal of unused medications. Veterinary drugs are widely regarded as environmental pollutants [4,11,12]; among them, antimicrobial agents arouse particular concern [13,14,15], with the main focus being on the development of antimicrobial resistance [14,16,17,18,19,20,21]. The presence of veterinary drugs in the environment is also associated with potential toxicity to off-target organisms [15,22] or changes in the population of nitrogen-fixing rhizobacteria occupying nodules of plants from the *Fabaceae* family [23].

Antiparasitic veterinary drugs constitute the second largest segment of the global animal health market, representing almost 25% of the total market share [24], yet the risks associated with their release to the environment from livestock and domestic animals have only recently become widely understood. Since then, studies have examined the transfer of antiparasitics, and other veterinary drugs, from manure to soil and their subsequent uptake by plants [6,8,25], edible insects [26], and earthworms, in the case of ivermectin [27].

Many of the studies performed on the environmental aspects of endoparasitic veterinary drugs, such as their bioaccumulation and ecotoxicity, have focused on avermectins [28,29,30,31]. However, there are also reports that milbemycins may have undesired environmental activity [31], as may albendazole [32,33], thiabendazole [34], fenbendazole, pyrantel and praziquantel [35], amprolium, bithionol, levamisole, and pyrimethamine [36]. Many of the drugs used against ectoparasites, such as fleas, ticks, or lice, are also potent, broad-spectrum, organophosphate, phenylpyrazole, neonicotinoid, or pyrethroid insecticides, or benzoylurea derivatives, used to protect crops [37,38,39,40]. They are toxic to several species, including off-target ones (as summarized in Table 1).

Antiparasitics have also been identified in milk from ruminants, and their stability during milk processing is a matter of rising concern [56,57,58,59].

Humans are exposed to veterinary drugs through direct contact and the consumption of contaminated food (meat, dairy products, eggs, bee products), but the scale of this exposure in still unknown in many countries [60]. While the health risks to humans presented by residual veterinary antiparasitics in foods are considered to be relatively low, assuming their correct administration, this data comes mainly from prolonged observations of their widespread use and records of compliance with the food residue standards [61]. In addition, the interactions of veterinary antiparasitic drugs with biological targets in humans are not fully understood, and new aspects of their toxicity and therapeutic applications are constantly being discovered [62,63].

An important fact influencing the bioactivity of a drug is its permeability across biomembranes [64,65]. Drugs are able to cross biomembranes, e.g., from the intestinal tract into the blood, or pass through the blood–brain barrier, through several mechanisms. These include transcellular uptake by passive diffusion (mainly small, moderately lipophilic/lipophilic molecules, e.g., aspirin), paracellular uptake (hydrophilic molecules such as desmopressin), transporter-mediated uptake (e.g., cefixime, valacyclovir), and by transcytosis (e.g., insulin) [64,66,67,68,69,70,71,72]. In some situations, transport is facilitated by both passive and carrier-mediated processes [73,74]. Cellular uptake of compounds is limited by their transporter-mediated efflux from cells, with one example being transport by P-glycoprotein (P-gp) [75].

Membrane permeability can be assessed in vitro using (i) cell-based models involving human colorectal adenocarcinoma (Caco-2) [69,73,76] or Madin–Darby canine kidney (MDCK) cells [77] and (ii) non-cell Parallel Artificial Membrane Permeability Assays (PAMPA) [78]. Caco-2 human colon epithelial cancer cells provide a relatively full mechanistic picture of passage across the intestinal barrier, including the associated permeability, efflux liability, and metabolic transformations; when cultured as a monolayer, they develop transporter proteins, efflux proteins, and Phase II conjugation enzymes, and hence are popular models of human intestinal absorption [70,76,79]. However, Caco-2 experiments are more time-consuming than other cell-based methods (e.g., MDCK assay) [77].

MDCK cells, similarly to Caco-2 cells, differentiate into columnar epithelium and form tight junctions when cultured on semipermeable membranes; as such, they are useful in studies of intestinal epithelial drug transport [77]. Cell-based methodologies are used to measure apparent permeability (***P***_app_), defined below [80]:

Papp= dQdtC0·A

where d***Q/***d***t***—the rate of permeation across the cells; ***C***_0_—donor concentration at time zero; ***A***—the area of the cell monolayer.

In PAMPA, donor compartments, i.e., those containing the studied compounds, are separated from acceptor compartments, i.e., those without the studied compounds, by filter plates pre-coated with synthetic phospholipid solutions in organic solvents. The permeation of compounds across the artificial phospholipid membrane is measured after incubation. This data is used to determine the effective permeabilities ***P***_e_ [81]:

$$P_{e}=C\cdot ln\left(1-\frac{{\left[drug\right]}_{acceptor}}{{\left[drug\right]}_{equilibrium}}\right)$$

$$C=\frac{V_{D}\cdot V_{A}}{\left(V_{D}+V_{A}\right)\cdot Area\cdot Time}$$

where ***V***_D_—volume of the donor compartment; ***V***_A_—volume of the acceptor compartment.

While PAMPAs exclusively mimic passive absorption and do not account for cellular factors like transporters or enzymes [78], they are very rapid and have lot of potential for modifications [82,83,84]. Different PAMPA tests can be used to determine the passage of compounds across different biological barriers, such as the skin, blood–brain barrier, or cornea [84,85,86,87,88,89,90].

The present study evaluates the impact of 86 veterinary antiparasitic drugs from different chemical families on the environment and human safety. It determines their membrane permeability, mobility in soil, potential to bioaccumulate in aquatic organisms (fish), oral absorption by humans, and mammalian blood–brain barrier permeability. The study uses novel QSAR models based on experimental reference data: blood–brain barrier permeability, bioconcentration factors in fish, and soil–water partition coefficients normalized to organic carbon. Its models employ easily calculated physico-chemical parameters of compounds and their theoretically predicted PAMPA and MDCK membrane permeabilities as independent variables. The results are intended to serve as the first stage in a systematic assessment of the potential risks to humans and the environment resulting from the widespread presence of veterinary antiparasitic drugs in the environment and the food chain.

## 2. Materials and Methods

### 2.1. Experimental Data

Experimental bioconcentration factors (log ***BCF***) for non-ionic compounds were compiled by Arnot and Gobas [91] into a database available via the EpiSuite software (BCFBAF v. 3.01) [92]. Experimental soil–water partition coefficients normalized to organic carbon (log ***K***_oc_) were taken from [93,94]. Both log ***BCF*** and log ***K***_oc_ reference datasets were revised, and compounds of dubious structures (mixtures of isomers, undefined isomers) and duplicates were removed; hence, 556 compounds with known log ***BCF*** and 632 compounds with known log ***K***_oc_ values remained. Unbound brain-to-plasma partition coefficients (***K***_p,uu_) for 74 compounds, obtained using a rat model, were reported by Lawrenz [95]. This rat reference data is considered to be in sufficient agreement with human data for use in pre-clinical screening of drugs [96,97]. Experimental log ***BCF***, log ***K***_oc_, and log ***K***_p,uu_ values are presented in the Supplementary Materials.

### 2.2. Calculated Molecular Descriptors and Membrane Permeability Data

Physico-chemical and ADMET properties were calculated using ADMETLab3.0 software (https://admetlab3.scbdd.com/, accessed on 30 September 2025) using SMILES strings extracted from PubChem as input data. The following physico-chemical descriptors were considered relevant to the studied biological properties: molecular weight (***MW***); van der Waals volume (***Vol***); Density = ***MW***/***Vol*** (***Dense***); count of hydrogen bond acceptors (***nHA***); count of hydrogen bond donors (***nHD***); number of rotatable bonds (***nRot***); number of rings (***nRing***); number of atoms in the biggest ring (***MaxRing***); number of non-carbon atoms (hydrogens included) (***nHet***); number of rigid bonds (***nRig***); flexibility = ***nRot***/***nRig*** (***Flex***); the logarithm of aqueous solubility value (log ***S***); the logarithm of the n-octanol/water partition coefficient (log ***P***); the logarithm of the n-octanol/water distribution coefficients at pH = 7.4 (log ***D***); number of sp^3^ hybridized carbons/total carbon count (***Fsp3***).

The following ADMET properties were considered in the study: theoretically predicted membrane permeabilities: log ***P***_app_ values (***caco2***; ***MDCK***) and the probability of a compound to be highly PAMPA-permeable (***PAMPA***); steady-state volume of distribution (log ***VD***ss); plasma protein binding, % (***PPB***); the fraction unbound in plasma, % (***Fu***). The ability of compounds to be absorbed in the gastro-intestinal tract was qualitatively evaluated using the SwissADME software (https://www.swissadme.ch/, accessed on 30 September 2025) [98] and quantitatively evaluated (***HIA***, %) using pkCSM software (https://biosig.lab.uq.edu.au/pkcsm/prediction, accessed on 30 September 2025) [99]. The values of the descriptors used in the study are listed in the Supplementary Materials.

### 2.3. Multiple Linear Regression (MLR) Models of log **K**_oc_, log **BCF** and **K**_p,uu,br_

Multiple linear regression models of log ***K****_oc_*, log ***BCF*** and log ***K***_p,uu,br_ were generated using Statistica v. 13.3 (StatSoft Kraków, Poland) in forward stepwise mode, using descriptors calculated by ADMETLab3.0 (Supplementary Materials). The descriptors were manually examined for collinearity using tolerance values. Tolerances were calculated as (1 − R^2^) and it was assumed that two descriptors were colinear if the tolerance value between them was <0.1 [100]. For every studied property, the models were validated using test sets of compounds, which were not used when generating the models. Compounds taken from previous studies [91,93,95] were randomly assigned to the training or test sets as follows (details given in Supplementary Materials):

- Log ***K***_oc_: training set—500; test set—132;
- Log ***BCF***: training set—400; test set—156;
- Log ***K***_p,uu_: training set—60; test set—14.

Multiple linear regression models were validated using R^2^, R^2^_adj._, and Q^2^ metrics for the training sets and RMSE_pred_ (Root Mean Square Error of Prediction) for the test sets [101].

### 2.4. Artificial Neural Network (ANN) Models of log **K**_oc_, log **BCF**, and **K**_p,uu,br_

Multilayer Perceptron (MLP) artificial neural networks (ANNs) were generated using Statistica v. 13.3 (regression, Automated Network Search—ANS module, with 1000 networks to train and 5 networks to retain). The neuron activation functions were selected from the following: identity, logistic, hyperbolic tangent, and exponential. The BFGS (Broyden–Fletcher–Goldfarb–Shanno) algorithm was used to train the network together with the sum of squares (SOS) or entropy error function. The quality of the ANN models was evaluated using correlation coefficients for the training, test, and validation sets to which the compounds taken from [91,93,95] were randomly assigned. The same independent variables used in the MLR models were also used in the ANN models. The significance of the independent variables in the ANN models was evaluated using global sensitivity analysis (GSA). This procedure rates the importance of an input variable in an ANN model by comparing the sums of the squared residuals for the model without the particular variable with those of the full model: when an input variable scores 1 or less than 1 in the GSA, this network is assumed to perform better without it. Detailed data on five retained networks for each studied parameter are given in the Supplementary Materials.

### 2.5. Boosted Tree (BT) Models of log **K**_oc_, log **BCF**, and **K**_p,uu,br_

Boosted Tree regression models were generated using Statistica v. 13.3 based on the same training and test datasets as those used to generate MLR models. The sets of independent variables used in the BT models were the same ones as used in MLR and ANN models, with the exception of log ***K***_p,uu_: in this case, log ***VDss*** was added after the manual examination of “predicted vs. observed” plots obtained for the modified sets of independent variables. The BT regression models were validated using R^2^ metrics for the training sets and RMSE_pred_ (Root Mean Square Error of Prediction) for the test sets.

## 3. Results

### 3.1. Prediction of Mobility in Soil

When studying pharmaceuticals, their mobility in soil is an important property, since their behavior in the soil–water compartment has a strong effect on both terrestrial and aquatic organisms [102,103]. Such mobility can be expressed by a soil–water partition coefficient. Since soils are complex and heterogeneous mixtures of minerals and organic matter, including humic substances, lipids and carbohydrates, the soil–water partition coefficient is usually normalized to organic carbon (***K***_oc_) to allow for comparison between different soils. These ***K***_oc_ values are used to assign compounds to soil mobility classes based on McCall’s or EPA criteria (Table 2) [104].

**Figure 1:**
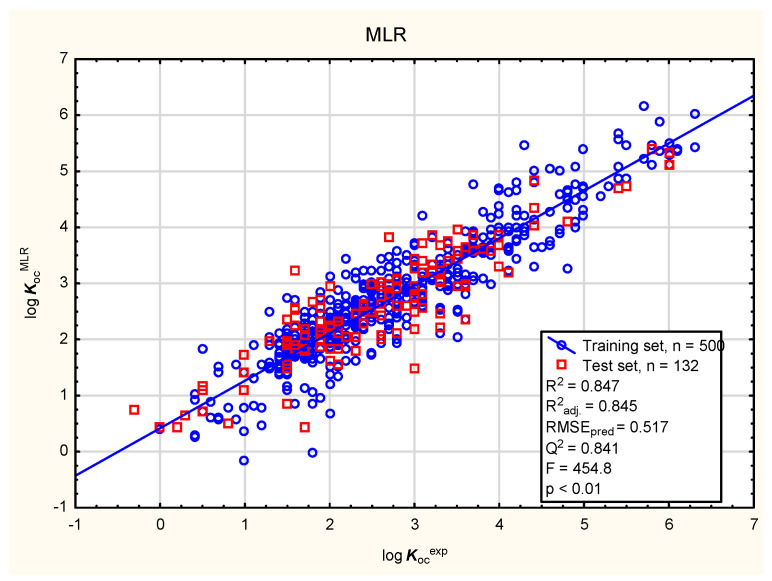
MLR model of log ***K***_oc_ (Equation (1))—predicted vs. observed (experimental) values.

**Figure 2:**
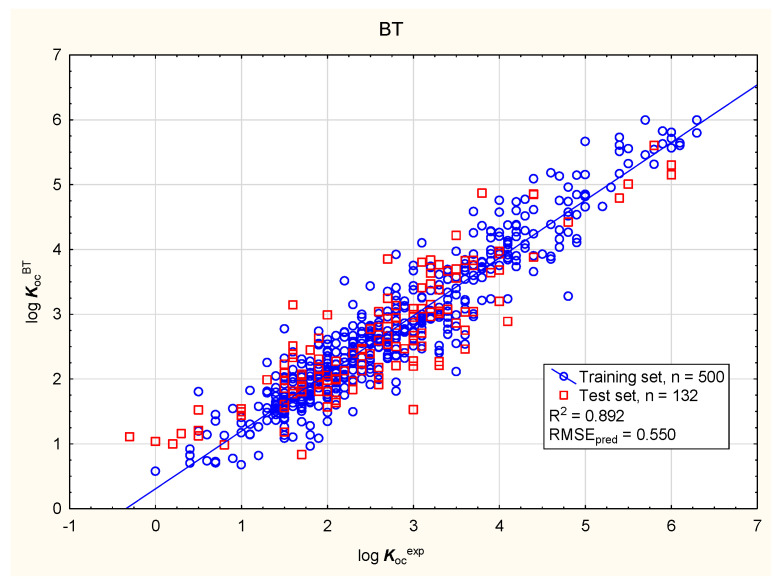
BT model of log ***K***_oc_—predicted vs. observed (experimental) values.

**Figure 3:**
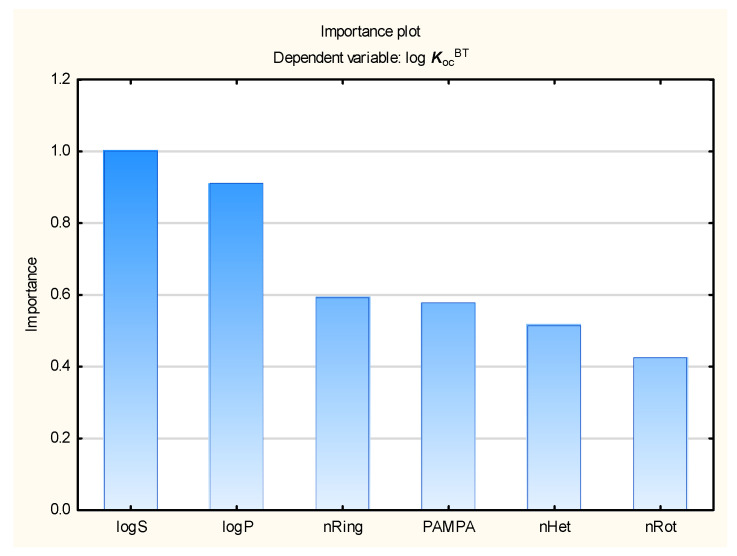
BT model of log ***K***_oc_—relative importance rating of descriptors.

**Figure 4:**
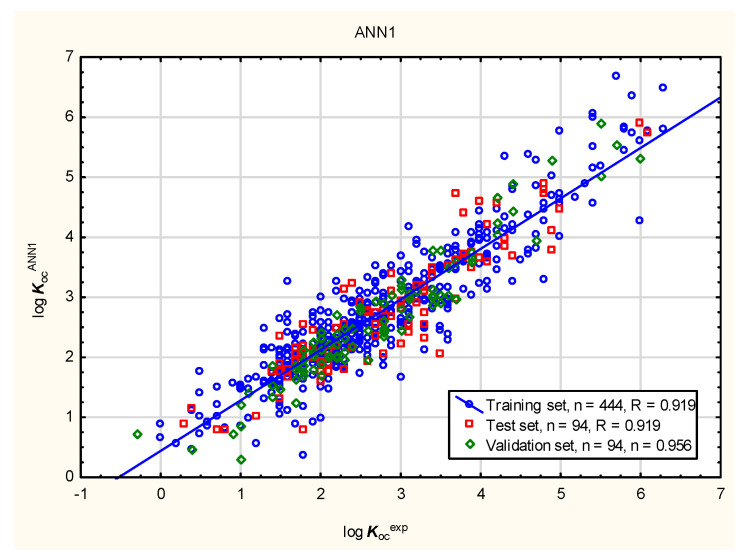
Sample ANN model of log ***K***_oc_—predicted vs. observed (experimental) values.

In the present study, the soil mobility of the tested antiparasitic drugs was determined using log ***K***_oc_ values; these were calculated using novel QSAR models based on a combination of simple physico-chemical properties and theoretically predicted membrane permeability descriptors. The models were obtained using MLR, BT, and ANN methods (Figure 1, Figure 2, Figure 3 and Figure 4; results for all 86 antiparasitic drugs in the Supplementary Materials).

Our data indicates that log ***K***_oc_ is inversely correlated with the ***PAMPA*** descriptor, solubility in water (log ***S***), and flexibility expressed as the count of rotatable bonds (***nRot***). In contrast, it is also positively correlated with lipophilicity (log ***P***) and heteroatom count (***nHet***), as shown in Equation (1):

log ***K***_oc_ = 0.921 (±0.067) − 0.0441 (±0.0091) ***nRot*** + 0.0309 (±0.0109) ***nHet*** + 0.152 (±0.029) ***nRing*** − 0.272 (±0.074) ***PAMPA*** − 0.201 (±0.033) log ***S*** + 0.356 (±0.034) log ***P*** (1)

### 3.2. Prediction of Bioconcentration in Aquatic Organisms

**Figure 5:**
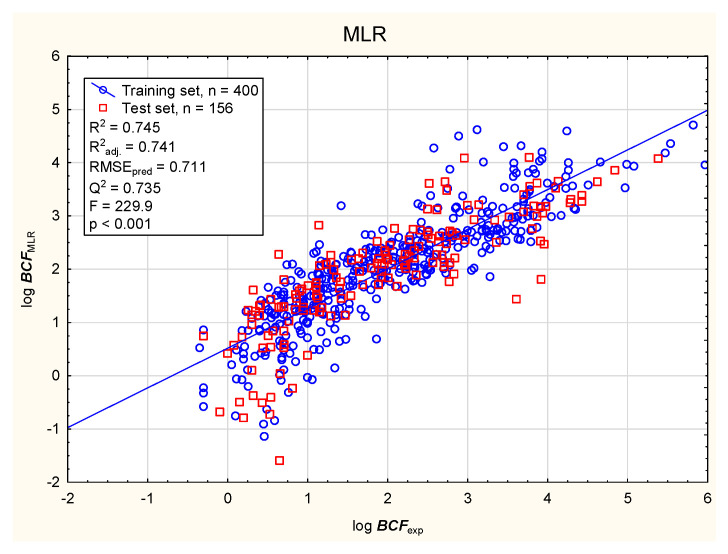
MLR model of log ***BCF***, Equation (2)—predicted vs. observed (experimental) values.

**Figure 6:**
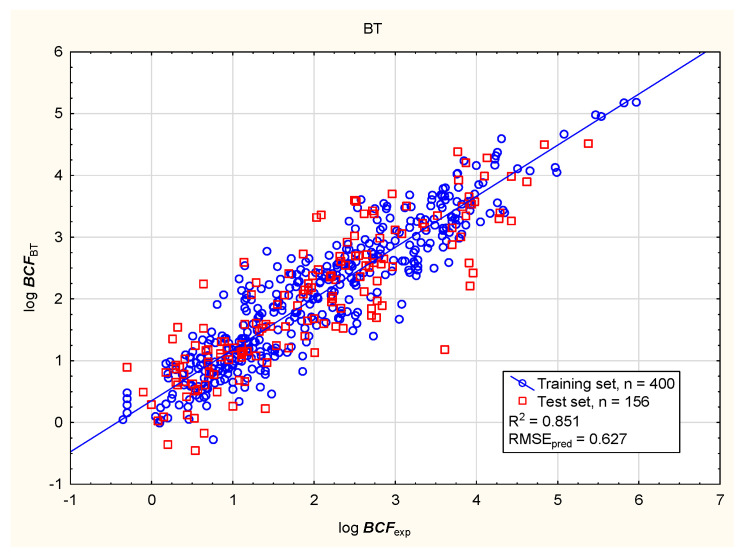
BT model of log ***BCF***—predicted vs. experimental values.

**Figure 7:**
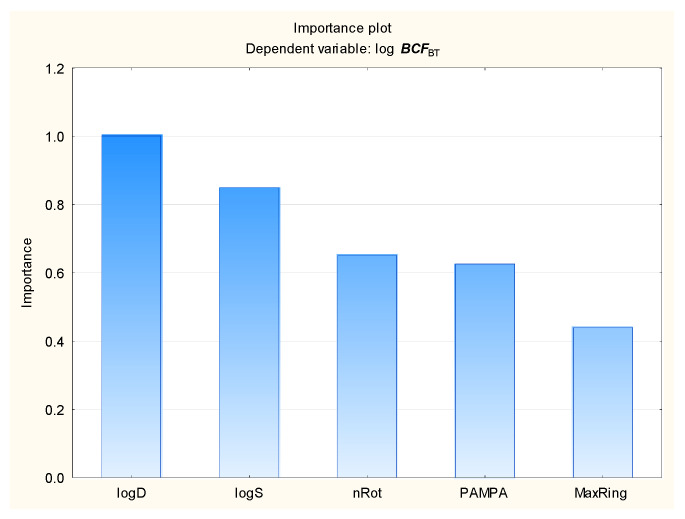
BT model of log ***BCF***—relative importance rating of descriptors.

**Figure 8:**
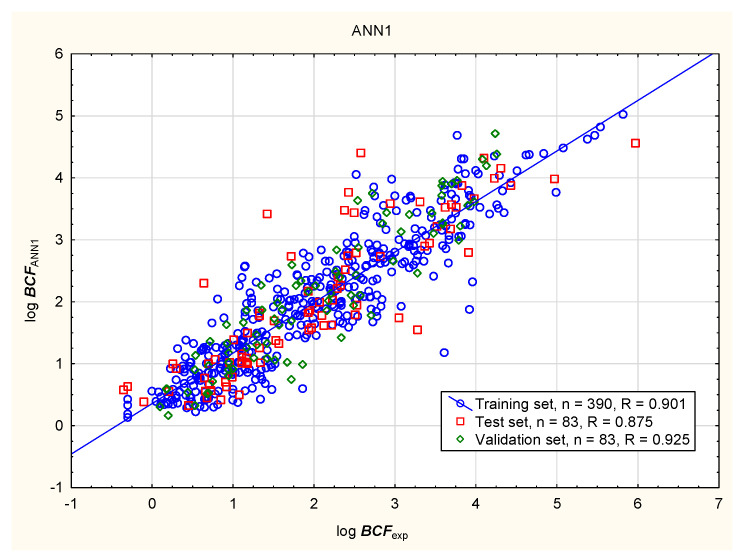
Sample ANN model of log ***BCF***—predicted vs. experimental values.

The intensive use of veterinary drugs in farming and in the treatment of pet animals may lead to the contamination of aquatic environments and the accumulation of drug residues in aquatic organisms, including fish, which may be consumed by humans. The fish bioconcentration factor (***BCF***) is the ratio of chemical concentration in the body of the fish to that in the surrounding water, to account for absorption via the respiratory tract and skin. The ***BCF*** is used in lieu of the bioaccumulation factor (***BAF***), which accounts for dietary, dermal, and respiratory absorption. The bioaccumulation criteria differ between regulatory bodies; however, it is assumed that compounds with log ***BCF*** > 3.7 or log ***BCF*** > 3.3 are capable of bioaccumulation [91]. The bioconcentration ability of the antiparasitic veterinary drugs was determined using the novel MLR, BT, and ANN models presented below: Equation (2), Figure 5, Figure 6, Figure 7 and Figure 8 (results in the Supplementary Materials).

log ***BCF*** = 0.668 (±0.133) − 0.130 (±0.011) ***nRot*** − 0.0901 (±0.0138) ***MaxRing*** − 0.543 (±0.128) ***PAMPA*** − 0.251 (±0.040) log ***S*** + 0.493 (±0.075) log ***D*** (2)

The log ***BCF*** values are inversely correlated with aqueous solubility (log ***S***), rotatable bond count (***nRot***), the largest ring size (***MaxRing***), and ***PAMPA***. They are also positively correlated with lipophilicity, expressed as log ***D*** at pH = 7.4

### 3.3. Prediction of Absorption from the Gastro-Intestinal Tract in Humans

Absorption of studied drugs from the human gastro-intestinal tract into the blood circulation was predicted using SwissADME and pkCSM software. According to the SwissADME predictions, 11 out of 86 antiparasitics are expected to be poorly absorbed from the GI tract (Supplementary Materials); however, the quantitative ***HIA*** absorption for all the studied drugs (including the externally used insecticides) is over the cut-off value of 30%, i.e., they are capable of being absorbed once ingested.

### 3.4. Prediction of the Blood–Brain Barrier Permeability

**Figure 9:**
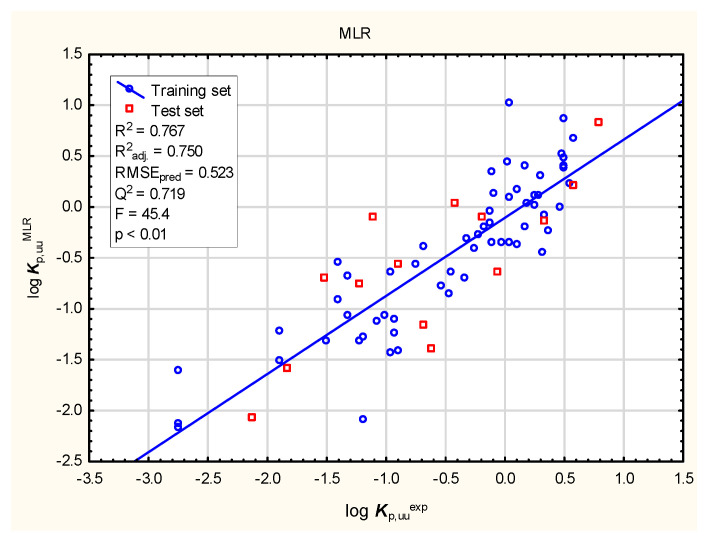
MLR model of log ***K***_p,uu_ (Equation (3))– predicted vs. observed (experimental) values.

**Figure 10:**
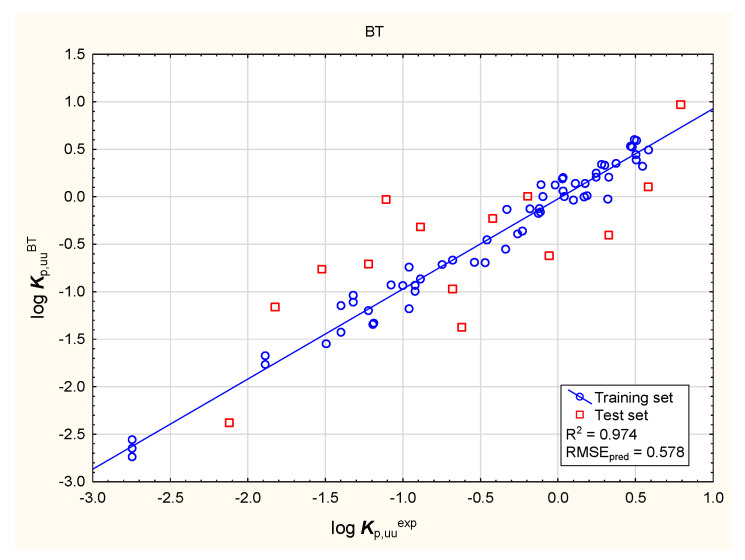
BT model of log ***K***_p,uu_—predicted vs. observed (experimental) values.

**Figure 11:**
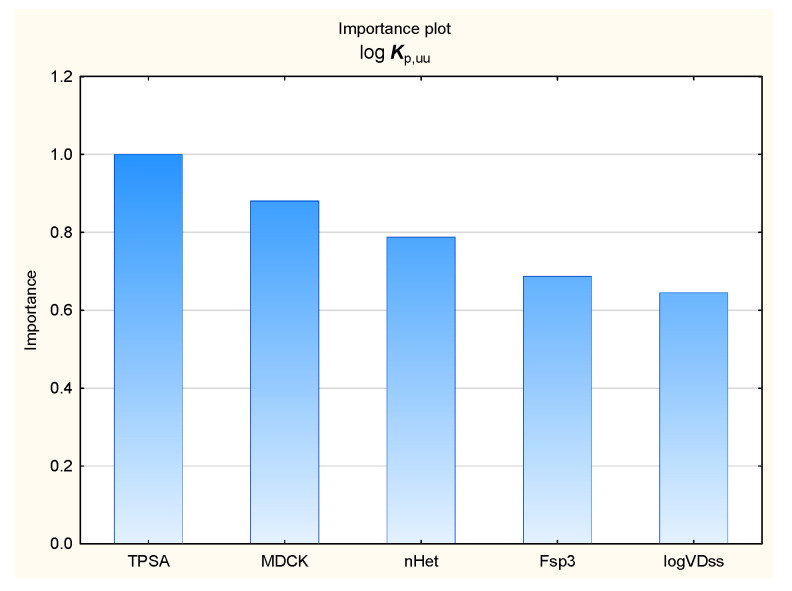
BT model of log ***K***_p,uu_—relative importance rating of descriptors.

**Figure 12:**
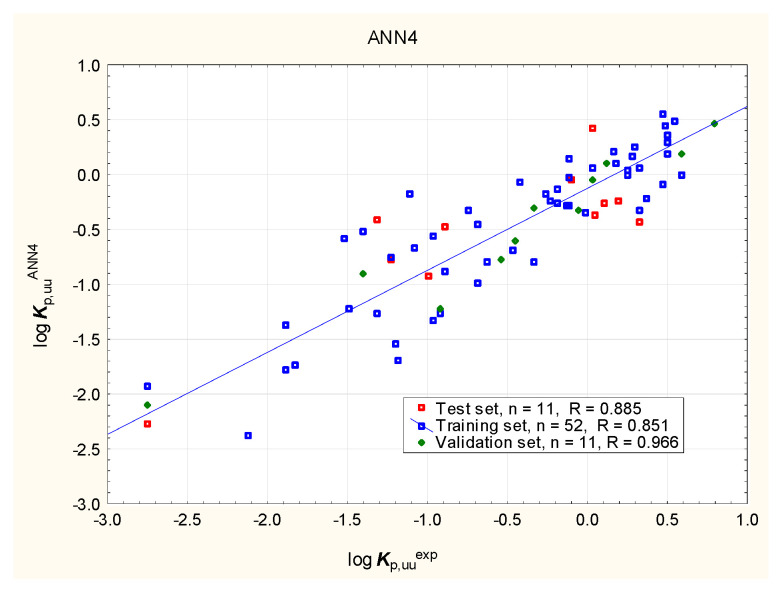
Sample ANN model of log ***K***_p,uu_—predicted vs. observed (experimental) values.

It is widely accepted now that excessive binding to plasma proteins can prevent compounds from interacting with biological targets in the brain. The ability of compounds to enter the central nervous system (CNS) may be expressed by the unbound brain-to-plasma concentration ratio (***K***_p,uu,brain_) or the unbound cerebrospinal fluid-to-plasma concentration ratio (***K***_p,uu,CSF_) [95,105,106]. Despite being less available, ***K***_p,uu_ descriptors are considered to be more clinically relevant than the ratio of the total drug concentration in the brain to that in the blood plasma in a state of equilibrium (***K***_p_ or ***BB***). The models of log ***K***_p,uu,br_ (denoted further as log ***K***_p,uu_) developed in this study (Equation (3) and Figure 9, Figure 10, Figure 11 and Figure 12) involve topological polar surface area (***TPSA***): according to Equation (3), this parameter is inversely correlated with log ***K***_p,uu_. Polar surface area is known to be an important predictor in several models of the total brain-to-blood concentration ratio (log ***BB***) [107]. According to Equation (3), ***TPSA*** seems, similarly, to influence the blood plasma-brain partition of unbound compounds. The log ***K***_p,uu_ value is also (positively) correlated with ***MDCK*** permeability, as indicated by Equation (3); other descriptors, including ***nHet*** and ***Fsp*^3^**, are less relevant.

log ***K***_p,uu_ = 6.81 (±1.37) − 0.0118 (±0.0034) ***TPSA*** − 0.0971 (±0.0373) ***nHet*** + 0.670 (±0.329) ***F***_sp3_ + 1.31 (±0.30) ***MDCK*** (3)

## 4. Discussion

### 4.1. Model Applicability

The models developed in this study are based on compounds with known experimental log ***K***_oc_, log ***BCF,*** and log ***K***_p,uu_ values. The key molecular/membrane permeability descriptors for the reference compounds were compared with those calculated for the antiparasitic drugs, with particular focus on the parameters used in the log ***K***_oc_, log ***BCF,*** and log ***K***_p,uu_ models developed herein:

- Log ***K***_oc_: ***nRot***, ***nHet***, ***nRing***, ***PAMPA***, log ***S*** and log ***P***;
- Log ***BCF***: log ***D***, log ***S***, ***nRot***, ***PAMPA***, ***MaxRing***;
- Log ***K***_p,uu_: ***TPSA***, ***MDCK***, ***nHet***, ***F***_sp3_, log***VD***_ss._

The values obtained for the studied antiparasitics largely overlap with those obtained for the reference compounds (Supplementary Materials).

**Figure 13:**
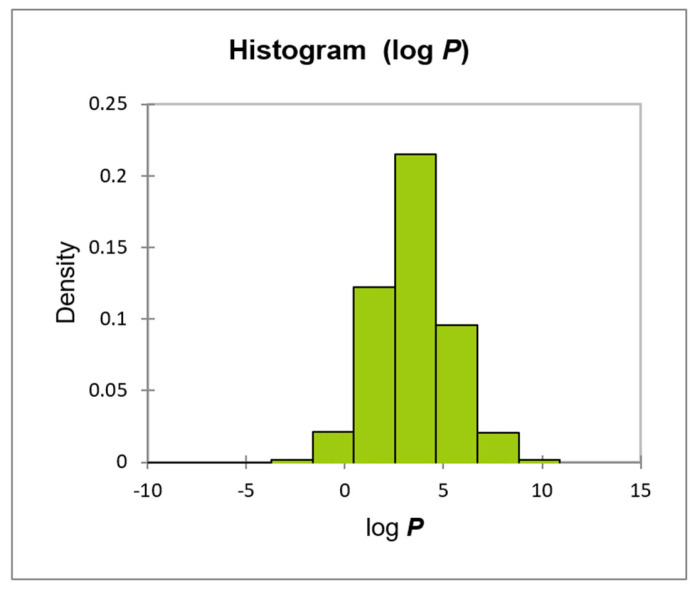
Histogram of log ***P*** values for reference compounds in log ***BCF*** models.

The majority of reported log ***BCF*** models based on lipophilicity are applicable to compounds with log ***P*** between ca. 1 and 6–7. This applicability can be extended by using different equations for different log ***P*** ranges, or by using non-linear models [108]. The log ***BCF*** models used in the present study were developed based on a large set of non-ionic compounds whose lipophilicity (log ***P***) ranged between −2.2 and +9.9 (Figure 13).

### 4.2. Analysis of the Predicted Properties in Different Chemical Families of Antiparasitics

**Figure 14:**
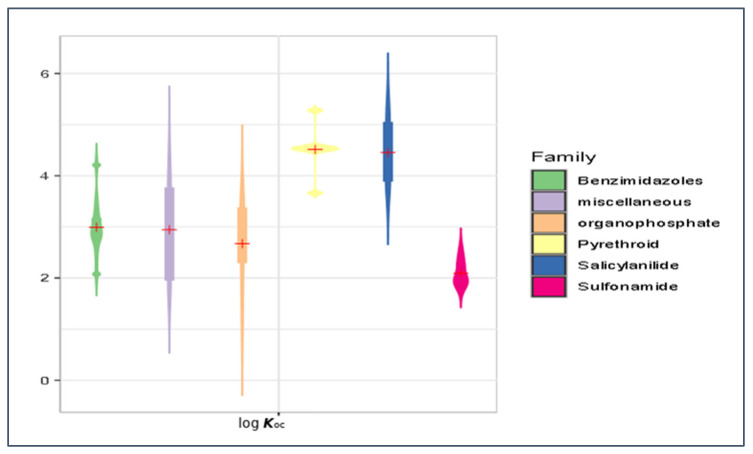
Violin plot of mean log ***K***_oc_ values for veterinary antiparasitic drugs.

**Figure 15:**
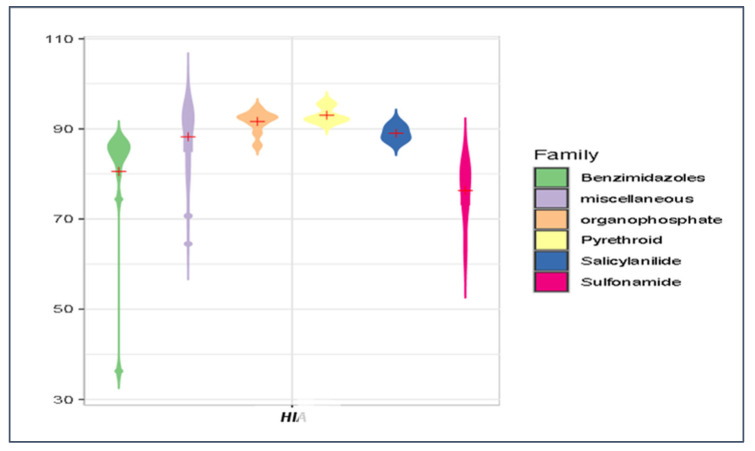
Violin plot of ***HIA*** values for veterinary antiparasitic drugs.

**Figure 16:**
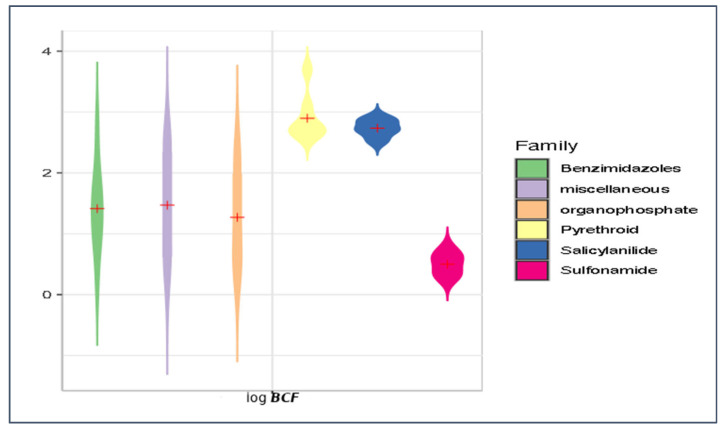
Violin plot of mean log ***BCF*** values for veterinary antiparasitic drugs.

**Figure 17:**
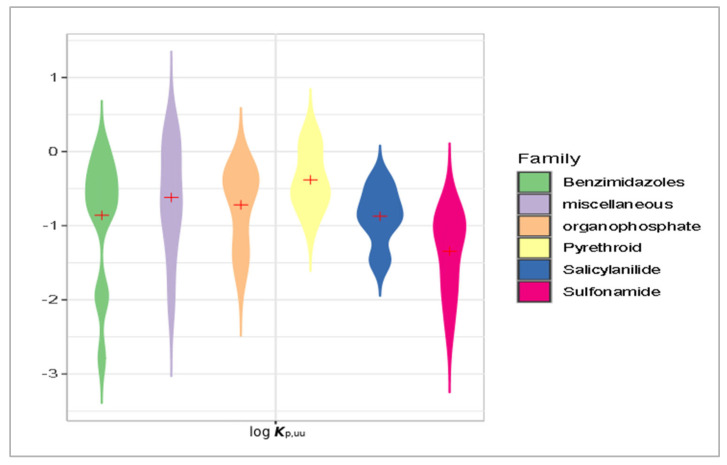
Violin plot of mean log ***K***_p,uu_ values for veterinary antiparasitic drugs.

Our predicted log ***K***_oc_, log ***BCF***, log ***K***_p,uu_, and ***HIA*** values were briefly compared for the main groups of antiparasitics (viz. benzimidazoles, organophosphates, pyrethroids, salicylanilides, and sulfonamides) using violin plots (Figure 14, Figure 15, Figure 16 and Figure 17). Based on the mean log ***K***_oc_, log ***BCF***, and log ***K***_p,uu_ values, the salicylanilides and pyrethroids are typically much less mobile in soil and demonstrate much higher bioconcentration in fish than sulfonamides. All tested compounds exhibited a similar potential to cross the blood–brain barrier. Organophosphates, pyrethroids, and salicylanilides are absorbed from the GI tract more readily than the other groups.

### 4.3. Comparison of Different Predictive Models

The log ***K***_oc_, log ***BCF***, and log ***K***_p,uu_ values calculated for the studied drugs were compared with the predictions obtained by the EpiSuite software [92] (for log ***K***_oc_ and log ***BCF***) and Equation (4), reported earlier [109] (for log ***K***_p,uu_), Table 3, Table 4 and Table 5.

log ***K*_p,uu,br_** = 0.866 − 0.211 **#*Heavy atoms*** − 0.250 **#*H-bond donors*** + 0.0272 ***MR*** + 0.483 ***iLOGP*** (4)

A high degree of similarity was observed between the log ***K***_oc_ or log ***BCF*** models developed in the present study; larger differences were noted between these models and the results produced by the EpiSuite software. The differences between the log ***K***_p,uu_ models proposed in this study are more pronounced.

### 4.4. Calculated vs. Experimental Values

Experimental log ***K***_oc_ or log ***BCF*** values are available only for small subsets of studied drugs: n = 15 for log ***K***_oc_ and n = 11 for log ***BCF*** [92]. The predicted log ***K***_oc_ and log ***BCF*** values for those subsets are in close agreement with the experimental data. Some of the log ***BCF*** and log ***K***_oc_ models (Table 6, underlined correlation coefficients) fit the experimental data slightly more closely than the data from the EpiSuite software, i.e., based on the Meylan methodology [110,111].

### 4.5. Drugs of Particular Concern

It was established that the majority of studied drugs are at least slightly mobile in soil. Three drugs, viz. closantel, rafoxanide, and flumethrin, were formally classified as “immobile”. No “highly mobile” drugs (log ***BCF*** < 1) were noted, but 19 appear “mobile” and 25 are “moderately mobile”. A detailed list of drugs whose mobility in soil suggests an elevated risk of groundwater pollution is given in the Supplementary Materials. “Mobile” and “moderately mobile” drugs were identified in all investigated groups, except for salicylanilides and pyrethroids.

Virtually all tested drugs were found to have a low risk of bioaccumulating in fish, with ***BCF*** values below the cut-offs quoted by Arnot and Gobas [91]. However, flumethrin demonstrated a mean predicted log ***BCF*** = 3.7 and is hence significantly more likely to bioaccumulate in fish.

Almost all tested drugs are drug-like with regard to Lipinski’s Ro5 (zero or maximum one violation). However, lufenuron slightly exceeds the crude limits for drug-like, orally available compounds (***MLOGP*** = 4.89 and ***MW*** = 511 vs. the cut-off values ***MLOGP*** = 4.15 and ***MW*** = 500 [112]).

**Figure 18:**
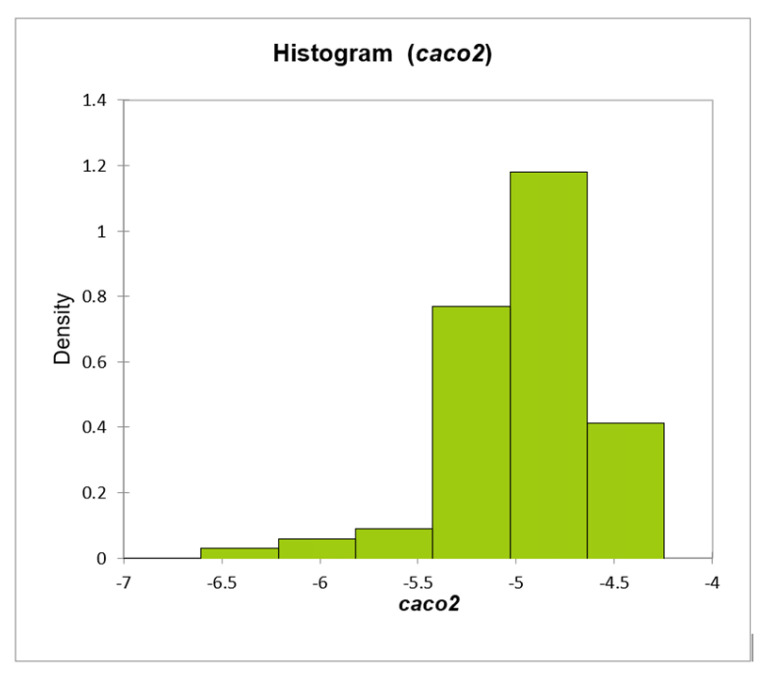
Distribution of calculated ***caco2*** values for 86 studied drugs.

For 18 of the studied drugs, the theoretically predicted Caco-2 permeabilities were found to be low, i.e., ***caco2*** below the cut-off value (–5.15 log units); detailed data in the Supplementary Materials and Figure 18. However, six of these compounds have predicted ***caco2*** values just below the threshold (i.e., –5.15 to –5.2). Five drugs from different chemical families (halofuginone, diminazen, pentamidine, amicarbalide, and chlorsulone) have ***caco2*** values below –5.5, suggesting impaired Caco-2 permeability.

Due to the considerable variation in the literature values, it is difficult to conclusively establish a cut-off value that could be used to identify compounds that are able to cross the blood–brain barrier. Nevertheless, it is generally assumed that a compound with ***K***_p,uu,brain_ > 0.3 to 0.5 (log ***K***_p,uu_ > −0.52 to −0.3) penetrates the brain easily [105]. Our findings indicate that 25 of the 86 drugs have relatively high log ***K***_p,uu_ (>−0.3) and 10 more have log ***K***_p,uu_ values between −0.3 and −0.52 (details in the Supplementary Materials). Five of the studied drugs (fipronil, amicarbalide, chlorsulone, febantel, and netobimin) demonstrated very low log ***K***_p,uu_ values (below −2); hence, their ability to enter the brain may be poor.

## 5. Conclusions

Our findings indicate that membrane permeability descriptors may be used in models aimed at predicting the environmental and pharmacokinetic properties of veterinary antiparasitic drugs, as well as other compounds. ***PAMPA*** does not play a major role as an independent variable in the soil–water partition (log ***K***_oc_) and fish bioconcentration (log ***BCF***) models; however, it was selected by stepwise regression in the MLR equations and it was found to have score higher than 1 in the ANN global sensitivity analysis, while in the BT models, it had a relative importance of 0.65 for log ***BCF*** and 0.57 for log ***K***_oc_.

In our blood–brain barrier permeability models, the membrane permeability descriptor ***MDCK*** was found to play an important role, selected at the second step of the stepwise MLR analysis, right after ***TPSA***; its relative importance in the BT regression is 0.88. It also demonstrated high scores in the ANN GSA, inferior only to those for ***nHet*** (with ***TPSA*** playing a less important role in the ANN models).

It was found that the majority of studied compounds are at least slightly mobile in soil. The studied drugs are absorbed by aquatic animals (e.g., fish) from the surrounding water, but are unlikely to bioaccumulate. Once ingested, they are likely to be absorbed from the gastro-intestinal tract; this is also possible for organophosphates and pyrethroids, which are primarily insecticides not intended for internal use. Finally, many of the studied drugs are capable of entering the mammalian (e.g., human) brain.

## Figures and Tables

**Table 1:** Studies on the toxicity of veterinary antiparasitics against off-target species.

Species	Drug	Refs.
Earthworms	Imidacloprid and dinotefuran	[38]
	Avermectins	[41,42]
Bees/pollinators	Pyrethroids	[43,44]
	Avermectins	[45]
	Selected acaricides used against Varroa	[46,47,48]
Aquatic invertebrates, e.g., Daphnia magna, Brachionus calyciflorus	Amprolium, bithionol, levamisole, and pyrimethamine	[36]
Aquatic macroinvertebrates	Flubendazole, fenbendazole, and ivermectin	[49]
Fish	Amprolium, bithionol, levamisole, and pyrimethamine	[36]
	Doramectin and flumethrin	[50]
	Pyriproxyfen	[51,52]
Birds	Ivermectin	[53]
	Fipronil, imidacloprid, and permethrin	[54]
	Toxicity to avian scavengers	[55]
Crustaceans	Pyriproxyfen	[52]

**Table 2:** Mobility in soil: EPA classification.

Range of log *K*_oc_	Mobility Class
<1	Very mobile
1–2	Mobile
2–3	Moderately mobile
3–4	Slightly mobile
4–5	Hardly mobile
>5	Immobile

**Table 3:** Pearson’s correlation coefficients between log ***K***_oc_ values calculated using different models for the 86 studied antiparasitic drugs.

n = 86	MLR	BT	ANN1	ANN2	ANN3	ANN4	ANN5	log ***K*_oc_^EPI^**
MLR	1.000	0.984	0.987	0.981	0.981	0.976	0.966	0.891
BT	0.984	1.000	0.970	0.966	0.963	0.956	0.952	0.870
ANN1	0.987	0.970	1.000	0.993	0.991	0.993	0.982	0.887
ANN2	0.981	0.966	0.993	1.000	0.989	0.993	0.975	0.877
ANN3	0.981	0.963	0.991	0.989	1.000	0.992	0.987	0.872
ANN4	0.976	0.956	0.993	0.993	0.992	1.000	0.981	0.880
ANN5	0.966	0.952	0.982	0.975	0.987	0.981	1.000	0.854
log ***K***_oc_^EPI^	0.891	0.870	0.887	0.877	0.872	0.880	0.854	1.000

**Table 4:** Pearson’s correlation coefficients between log ***BCF*** values calculated using different models for 86 antiparasitics.

n = 86	MLR	BT	ANN1	ANN2	ANN3	ANN4	ANN5	log ***BCF***_EPI_
MLR	1.000	0.953	0.978	0.962	0.976	0.973	0.974	0.822
BT	0.953	1.000	0.966	0.962	0.956	0.963	0.965	0.814
ANN1	0.978	0.966	1.000	0.990	0.995	0.995	0.996	0.837
ANN2	0.962	0.962	0.990	1.000	0.987	0.994	0.990	0.836
ANN3	0.976	0.956	0.995	0.987	1.000	0.997	0.996	0.828
ANN4	0.973	0.963	0.995	0.994	0.997	1.000	0.997	0.838
ANN5	0.974	0.965	0.996	0.990	0.996	0.997	1.000	0.833
log ***BCF***_EPI_	0.822	0.814	0.837	0.836	0.828	0.838	0.833	1.000

**Table 5:** Pearson’s correlation coefficients between log ***K***_p,uu_ values calculated using different models for the 86 studied antiparasitic drugs.

n = 86	MLR	BT	ANN1	ANN2	ANN3	ANN4	ANN5	log ***K***_p,uu_^(4)^
MLR	1.000	0.856	0.913	0.955	0.948	0.841	0.910	0.870
BT	0.856	1.000	0.830	0.864	0.863	0.772	0.814	0.825
ANN1	0.913	0.830	1.000	0.981	0.983	0.978	0.961	0.777
ANN2	0.955	0.864	0.981	1.000	0.998	0.931	0.955	0.809
ANN3	0.948	0.863	0.983	0.998	1.000	0.932	0.943	0.807
ANN4	0.841	0.772	0.978	0.931	0.932	1.000	0.950	0.713
ANN5	0.910	0.814	0.961	0.955	0.943	0.950	1.000	0.768
log ***K***_p,uu_^(4)^	0.870	0.825	0.777	0.809	0.807	0.713	0.768	1.000

**Table 6:** Pearson’s correlation coefficients for log ***BCF*** and log ***K***_oc_.

n = 11	MLR	BT	ANN1	log ***BCF***_EPI_	log ***BCF***_exp_	n = 15	MLR	BT	ANN1	log ***K*_oc_**^EPI^	log ***K*_oc_**^exp^
MLR	1.000	0.974	0.966	0.843	0.912	MLR	1.000	0.990	0.995	0.954	0.956
BT	0.974	1.000	0.943	0.892	0.905	BT	0.990	1.000	0.975	0.924	0.932
ANN1	0.966	0.943	1.000	0.855	0.882	ANN1	0.995	0.975	1.000	0.956	0.970
log ***BCF***_EPI_	0.843	0.892	0.855	1.000	0.845	log ***K***_oc_^EPI^	0.954	0.924	0.956	1.000	0.944
log ***BCF***_exp_	0.912	0.905	0.882	0.845	1.000	log ***K***_oc_^exp^	0.956	0.932	0.970	0.944	1.000

## Data Availability

The original contributions presented in the study are included in the article. Further inquiries can be directed to the corresponding author.

## Supplementary Materials

The following supporting information can be downloaded at: https://www.mdpi.com/article/10.3390/membranes16010039/s1. Table S1: Reference compounds for log ***K***_oc_ models; Table S2: Correlations between the properties of reference compounds; Table S3: Predicted membrane permeabilities (***caco2***, ***MDCK***, ***PAMPA***), ***HIA*** absorption (%), log ***K***_oc_, log ***BCF*** and log ***K***_p,uu_ for 86 antiparasitic drugs; Table S4: mobility in soil classification according to EPA and binary gastro-intestinal (GI) absorption classification; Table S5: Reference compounds for log ***K***_p,uu_ models; Table S6: ANN validation; Table S7: Reference compounds for log ***BCF*** models; Table S8: Properties of 86 drugs; Figure S1: Log ***K***_p,uu_-Comparison of key properties of the reference set (n = 74) and studied compounds (n = 86); Figure S2: Log ***K***_oc_-Comparison of key properties of the reference set (n = 632) and studied compounds (n = 86); Figure S3: Log ***BCF***—Comparison of key properties of the reference set (n = 556) and studied compounds (n = 86).
